# Reply: “Vitamin D Supplementation in Influenza and COVID-19 Infections. Comment on: Evidence That Vitamin D Supplementation Could Reduce Risk of Influenza and COVID-19 Infections and Deaths *Nutrients* 2020, *12*(4), 988”

**DOI:** 10.3390/nu12061620

**Published:** 2020-06-01

**Authors:** William B. Grant, Carole A. Baggerly, Henry Lahore

**Affiliations:** 1 Sunlight, Nutrition, and Health Research Center, P.O. Box 641603, San Francisco, CA 94164-1603, USA; 2 GrassrootsHealth, Encinitas, CA 95101, USA; carole@grassrootshealth.org; 3 2289 Highland Loop, Port Townsend, WA 98368, USA; hlahore@gmail.com

**Keywords:** African Americans, COVID-19, C-reactive protein, hemoglobin, influenza, randomized controlled trials, respiratory tract infections, vitamin D, vitamin D_2_, vitamin D_3_

We thank Dr. Hasan for the comments [1] on our review [2] and for providing us the opportunity to extend the discussion regarding the role of vitamin D in reducing the risk of respiratory tract infections.

Dr. Hasan’s first point: “However, we are rather concerned with authors’ recommendation that people at risk of COVID-19 should consider ‘taking 10,000 IU/d of vitamin D_3_ for a few weeks to rapidly raise 25(OH)D concentrations, followed by 5000 IU/d to reduce the risk of infection’. We believe that authors’ recommendation of using a high dose of vitamin D supplementation is inappropriate as there is no robust clinical evidence to support such claims.” Our recommendation was based on reaching a serum 25-hydroxyvitamin D [25(OH)D] concentration between 40 and 60 ng/mL in advance of the winter viral respiratory tract infection (RTI) season. According to Figure 1 in an article by Heaney [3], when starting at a level of around 20 ng/mL it takes about 35 days to reach 60 ng/mL with 10,000 IU/d and 85 days with 4000 IU/d. A randomized controlled trial (RCT) published in 2015 showed that after a single dose of 250,000 IU of vitamin D_3_ given to healthy volunteers between the ages of 18 and 65 years with baseline serum levels of <17 ng/m, serum 25(OH)D concentrations at five days increased to an average of 41 ng/mL [4]. There were no adverse effects. However, after 90 days, 25(OH)D concentrations were back to near baseline values. In the preprint of the first submitted version of our review [5], we discussed the results of reported influenza-like illness (ILI) with respect to serum 25(OH)D concentrations for GrassrootsHealth participants (this information was omitted from the published review since it should go through the peer review process independent of its inclusion in a review.) Table 1 reports that, compared to [25(OH)D] of <20 ng/mL, the adjusted odds ratio for ILI for 40–49 ng/mL, 50–59 ng/mL, and ≥60 ng/mL were 0.04, 0.02, and 0.03, respectively. While this finding was not related to COVID-19, there are indications that viral RTIs have similar etiologies. Those who are concerned about exposure to the COVID-19 virus or who have symptoms of COVID-19 infection might benefit from a much larger dose, although clinical trials need to be performed.

Very recently, a preprint reported severity of COVID-19 infections with respect to 25(OH)D concentration in three Asian countries [6]. Out of 49 patients with mild symptoms, 47 had serum 25(OH)D concentrations of >30 ng/mL compared with only four of 59 with ordinary symptoms, two of 56 severe patients, and two of 48 critical patients. The mean serum 25(OH)D concentrations for mild, ordinary, severe, and critical patients were 31, 27, 21, and 17 ng/mL, respectively. 

Results of a study of seroprevalence for COVID-19 in Santa Clara County, California were reported in a preprint [7], with A total of 3330 people included; 50 people were found to have antibodies for COVID-19. After adjusting the data to correspond to the demographic characteristics of the county, the seroprevalence to COVID-19 was estimated at between 2.49% and 4.16%, with uncertainty bounds ranging from 1.80% up to 5.70%. This estimate translates to 48,000 to 81,000 people in the county, which is 50 to 80 times the 956 people that were identified by April 1, 2020. It should be noted that this study has not been peer reviewed and may have some methodological issues to address. We think it likely that many have contracted COVID-19 with minimal or no symptoms. Supporting this idea, several coronaviruses exist that result in minor colds in the January–February timeframe, as found from a study of children crossing the Southern China–Hong Kong border [8]. 

These two studies support our suggestion of 40 to 60 ng/mL, since one study presented COVID-19 infection with 25(OH)D concentrations of >30 ng/mL and the other found that many people were infected without symptoms. Elderly people with chronic diseases are very likely to have low 25(OH)D concentrations, as discussed in our review [2]. However, further studies are required to better determine the threshold for protection against COVID-19 infection with symptoms.

As is well known, COVID-19 patients in critical condition often require a ventilator to help supply oxygen to their blood. A meta-analysis of laboratory findings of clinical characteristics for COVID-19 patients found that the pooled frequency of anemia from two studies was 44% (95% CI, 30%–58%), while the pooled frequency for high C-reactive protein (CRP) from eight studies was 72% (95% CI, 54%–85%) [9]. A study conducted in Egypt found that serum 25(OH)D concentration was inversely correlated with the degree of severity of acute lower RTIs (*r* = 0.80) in hospitalized infants with a mean age of 11 ± 3 months [10]. In addition, hemoglobin level was also highly correlated with serum 25(OH)D concentration (*r* = 0.71), with the mean concentration ranging from 9 ng/dL for 6 ng/mL 25(OH)D to 14 ng/dL for 50 ng/mL 25(OH)D. 

While low-dose vitamin D supplementation was not found to increase hemoglobin concentration in short-term studies [11], high-dose vitamin D supplementation was. A clinical trial involving 30 mechanically ventilated, critically ill adults were assigned to three groups to receive a placebo, 250,000 IU vitamin D_3_, or 500,000 IU vitamin D_3_ total during a five-day periods [12]. Mean baseline hemoglobin concentration was between 8.5 and 10.5 g/dL for the three groups. Hemoglobin concentration increased significantly only for the 500,000 IU vitamin D_3_ group, who experienced a 2 g/dL increase in four weeks. However, a phase 3 RCT involving 1078 critically ill vitamin D-deficient patients, with those in the treatment arm given 540,000 IU vitamin D_3_ supplementation within 12 h of admission to an intensive care unit, found no significant benefit in terms of 90-day mortality rate (*P* = 0.26) or with respect to secondary clinical, physiological, or safety end-points [13]. 

A cross-sectional study using data from the USA’s National Health and Nutrition Examination Surveys data found that CRP varied from 222 (95% CI, 205–241) mcg/dL for a 25(OH)D concentration of <12 ng/mL to 199 (179–201) mcg/dL for >30 ng/mL 25(OH)D [14]. 

Thus, by analogy, vitamin D deficiency appears to be a very important risk factor for severe COVID-19 infection. 

Dr. Hasan’s second point: “The authors have conveniently ignored the results of some key clinical studies evaluating the effectiveness of vitamin D supplementation in reducing the risk of developing respiratory tract infections (RTIs).” The meta-analysis of 15 RCTs on the effectiveness of vitamin D supplementation on risk of RTIs by Gysin et al. [15] had a serious flaw: the evaluations were made based on vitamin D dose vs. placebo, not serum 25(OH)D concentration. Vitamin D does not have a direct bearing on disease risk; it is 25(OH)D concentration that was found to be associated with disease risk. Inspection of the RCTs used in their analysis in Figure 3 presented some with very low baselines with significantly reduced risk of RTIs and very low vitamin D doses, while others with high baseline 25(OH)D concentrations and high vitamin D doses exhibited no effect. Heaney’s guidelines for RCTs for nutrients such as vitamin D were discussed in our review [16], the most important factor being that they be based on 25(OH)D concentration, both baseline and achieved, and that sufficient vitamin D_3_ be given.

Regarding the individual participant data meta-analysis of RTI in vitamin D RCTs [17], there were few participants who achieved a 25(OH)D concentration of >40 ng/mL, so they could not adequately assess the impact of high 25(OH)D concentration. We noted that a study in Connecticut found 38 ng/mL as the threshold for a significantly lower risk of community-based pneumonia [18].

Dr. Hasan’s third point: “Although high dose vitamin D3 was not found to increase the risk of kidney stone or hypercalcemia, it is not devoid of side effects as a randomized clinical trial only observed significant lower radial bone and tibial bone mineral density with 3-year-treatment of vitamin D at a dose of 10,000 IU/d”. Regarding the possible adverse effects of high-dose vitamin D supplementation, we read the article by Burt et al. [19]. As mentioned in the comment, the only adverse finding was a reduction (3.5%) in bone mass density. However, bone mass density does not equate to bone strength, and the reductions in strength measured as failure load were not significant. That was a three-year study, whereas we are suggesting a strategy for the winter RTI season. We also note that all pharmaceutical drugs have adverse side effects. If high-dose vitamin D is considered a drug, it differs from pharmaceutical drugs in that it has many side benefits [20].

Dr. Hasan’s fourth point: “Given the possible negative impact on bone mineral density with high dose vitamin D3, it is probably wise to wait for the results of ongoing clinical trials that are registered to explore the relationship between vitamin D and COVID-19.” We agree that RCTs should be conducted to evaluate the role of vitamin D in preventing and treating COVID-19 infection. However, we strongly disagree that vitamin D supplementation should be held in abeyance for prevention until such RCTs are completed and reported. Those at highest risk of infection due to having chronic disease, low 25(OH)D status, and/or being in frequent contact with others likely to be infected should be taking vitamin D. As noted, there is mounting evidence that vitamin D can reduce the risk and severity of RTIs, including that the mechanisms are known, that there are many health benefits of higher 25(OH)D concentrations, and that there are very few adverse effects of vitamin D_3_ supplementation. Vitamin D has demonstrated effectiveness in reducing the risk of overall cancer incidence and death, as well as the risk of progressing from pre-diabetes to diabetes in secondary results of major vitamin D RCTs [21]. Thus, there is much to gain and little to lose by taking vitamin D supplements now for COVID-19 prevention. RCTs should be conducted for treatment to explore at which stages of infection what baseline 25(OH)D concentrations, vitamin D doses, and achieved 25(OH)D concentrations are associated with benefits and adverse effects, if any.

Dr. Hasan’s fifth point questioned our statement: “A clinical trial involving postmenopausal women living on Long Island, NY with mean baseline 25(OH)D concentration 19 ± 8 ng/mL found that supplementation with 2000 IU/d resulted in significantly fewer upper respiratory tract infections, including influenza, than a placebo or supplementation with 800 IU/d [22].” In this trial, 104 participants took a placebo for three years and suffered from 29 RTIs in total, 104 took 800 IU/d vitamin D_3_ for two years and suffered 8 RTIs, and 104 took 2000 IU/d vitamin D_3_ and one RTI was recorded. The odds ratio for 800 IU/d vs. placebo was 0.39 (95% confidence interval, 0.17–0.87, *P* = 0.02), while that for 2000 IU/d vs. placebo was 0.09 (0.01–0.50, *P* = 0.02).

We use this opportunity to respond to two important comments by other readers of our review. One questioned why we did not indicate that vitamin D_3_ (cholecalciferol) be used rather than vitamin D_2_ (ergocalciferol), the answer being that, in some countries, the only high-dose vitamin D is ergocalciferol. However, cholecalciferol is a better choice, in part since it is the type of vitamin D produced in the skin through ultraviolet B irradiation of 7-dehydrocholesterol followed by a thermal reaction. After publication of our review, an article was published reporting the effects of vitamin D on gene expression of rat oligodendrocyte precursor cells. The study found that vitamin D_3_ influenced 1272 genes in 24 h compared to only 574 for vitamin D [23]. Most of the effects of vitamin D are through the hormonal metabolite 1,25-dihydroxyvitamin D, which activates vitamin D receptors bound to chromosomes, thereby affecting the expression of many genes.

The second question was why we did not point out that African Americans (AAs) have a much higher risk of COVID-19 infection and death than white Americans. At the time we submitted our manuscript, the data comparing AA COVID-19 infection and mortality rates were not available. In addition, there are a number of other reasons why AAs have higher COVID-19 rates, including that they have higher chronic disease rates than white Americans [24]. 

People with chronic diseases generally have low 25(OH)D concentrations (see Table 2 in [2]). Now, however, it is well-known that AAs have much higher COVID-19 infection and mortality rates [25]. Based on the National Health and Nutrition Examination Survey (NHANES) 2001–2010, the prevalence of serum 25(OH)D concentrations <20 ng/mL was 72% for non-Hispanic blacks (NHBs), 43% for Hispanics, and 19% for non-Hispanic whites, with the prevalence of <10 ng/mL being 17% in NHBs [26]. Of all the risk factors AAs have for becoming infected with COVID-19, raising serum 25(OH)D concentrations is the easiest one to counter.
